# Highly Sensitive Molecular Diagnostic Platform for Scrub Typhus Diagnosis Using *O. tsutsugamushi* Enrichment and Nucleic Acid Extraction

**DOI:** 10.3390/bios14100493

**Published:** 2024-10-10

**Authors:** Myoung Gyu Kim, Seulki Kim, Juho Jang, Jinkwan Lee, Namheon Kim, Yeji Yu, A Reum Kim, Seungjin Lim, Moonsuk Bae, Yong Shin

**Affiliations:** 1 Department of Biotechnology, College of Life Science and Biotechnology, Yonsei University, 50 Yonsei-ro, Seodaemun-gu, Seoul 03722, Republic of Korea; kmg941104@yonsei.ac.kr; 2 Division of Infectious Diseases, Department of Internal Medicine, Pusan National University Yangsan Hospital, Yangsan 50561, Republic of Korea; seul8806@pnuyh.co.kr (S.K.); yj919@pnuyh.co.kr (Y.Y.); id1626@pnuyh.co.kr (A.R.K.); babopm@pusan.ac.kr (S.L.); 3 Research Institute for Convergence of Biomedical Science and Technology, Pusan National University Yangsan Hospital, Yangsan 50561, Republic of Korea; 4 INFUSIONTECH, 38 Heungan-daero, 427 Beon-gil, Anyang-si 14059, Republic of Korea; jhjang@infusiontech.co.kr (J.J.); jklee@infusiontech.co.kr (J.L.); nhkim@infusiontech.co.kr (N.K.); 5 Department of Internal Medicine, Pusan National University School of Medicine, Busan 46241, Republic of Korea

**Keywords:** scrub typhus, molecular diagnostics, sample preparation, DNA detection kit

## Abstract

Scrub typhus is caused by the Gram-negative obligate intracellular bacterium *Orientia tsutsugamushi*, and this tick-borne disease is difficult to distinguish from other acute febrile illnesses as it typically presents with symptoms such as rash, crusting at the bite site, headache, myalgia, lymphadenopathy, and elevated liver transaminases. It can often be diagnosed clinically, but not all patients present with characteristic symptoms, so serological diagnosis and molecular techniques may be required. However, existing diagnostic tests often have low sensitivity and specificity, making early detection difficult. This study presents a nucleic acid extraction method using large volumes of plasma and buffy coat to increase sensitivity, as well as an improved detection method using two target genes. Using the I-PULL device, nucleic acids can be extracted from up to 4 mL of sample in 30 min, avoiding contamination. The extracted DNA detects two genes of *O. tsutsugamushi*, increasing sensitivity compared to single-gene detection. Clinical validation in 38 patient samples showed 100% specificity and 95.24% sensitivity for the single target gene, with specificity and sensitivity rising to 100% when both genes are analyzed. This molecular diagnostic platform can be useful for distinguishing scrub typhus from similar diseases.

## 1. Introduction

Scrub typhus is caused by *Orientia tsutsugamushi*, a Gram-negative obligate intracellular bacterium, and it was originally considered as a major public health problem in endemic areas known as the Tsutsugamushi triangle [1]; however, it is extending beyond these traditional boundaries [2,3]. It is a mite-borne infectious disease and typically presents as an acute febrile illness with rash, eschar at the bite site, headache, myalgia, generalized lymphadenopathy, and elevation of liver transaminases, which cannot be differentiated from symptoms of other acute febrile diseases [4], such as dengue fever, malaria, chikungunya, and leptospirosis [5,6]. Moreover, the clinical presentation of other tick-borne infections such as severe fever with thrombocytopenia syndrome, anaplasmosis, and Q fever can mimic symptoms of scrub typhus, making distinguishing them challenging [7]. Because these infectious diseases have different treatment strategies, disease courses, and clinical prognoses [8,9,10], it is essential to differentiate scrub typhus from other etiologies of acute febrile disease rapidly and accurately.

Diagnosis of the disease can usually be made clinically by identifying characteristic findings such as scabs and rashes along with a history of outdoor activity. It can be difficult to diagnose based on clinical symptoms alone, as not all patients present with a rash or scabs. In these cases, serologic diagnostics, gene detection, and culture testing are used. However, currently developed diagnostic tests have low sensitivity and specificity, which makes it difficult to diagnose the disease early due to false positive and false negative results. Currently, several molecular diagnostic techniques are being developed for the early detection of *O. tsutsugamushi*. A polymerase chain reaction (PCR)-based diagnostic testing strategy to identify *O. tsutsugamushi* has been increasingly applied to diagnose scrub typhus with the advantages of early diagnosis [11,12]. However, because of its intracellular habitat and low concentration in whole blood, detecting specific DNA for *O. tsutsugamushi* by conventional PCR has a low-sensitivity problem [13]. This sensitivity has been enhanced by using a mixture of buffy coat and plasma instead of whole blood as the sample for genomic DNA extraction. According to previous studies, the buffy coat bacterial loads were approximately 10-fold higher than in whole blood samples [14]. In addition, adding plasma to buffy coat can maximize the harvest of microbial DNA during the DNA extraction step and concentrate microbial DNA in the final eluted buffer, improving sensitivity [15].

Most of these diagnostic assays focus on the detection methods, and reports on sample preparation are still scarce. The process of extracting nucleic acids from pathogens for molecular diagnosis requires the use of centrifuges and solid-phase silica membranes. However, these extraction methods have various limitations, such as contamination and pathogen spread and loss, the requirement of sophisticated instrumentation and expensive reagents, and labor-intensive processes. To overcome these limitations, recent research has focused on rapid separation methods using microfluidic chips, metal materials, and polymer materials [16,17,18]. However, the concentration of *O. tsutsugamushi* in blood is extremely low in the early stages of the disease; the use of large sample volumes during the sample preparation step is crucial as it is highly correlated with the sensitivity of *O. tsutsugamushi* detection in clinical specimens. Above all, highly specific and sensitive diagnostic methods including sample preparation and detection techniques are needed for early diagnosis and effective monitoring of scrub typhus.

In this study, a molecular diagnostic platform was developed using a large volume of plasma and buffy coat with which to improve the low sensitivity of the scrub typhus diagnostic method. We present a nucleic acid extraction method (I-PULL) that can be used as a point-of-care diagnostic system and a detection method for highly sensitive diagnosis of scrub typhus. The I-PULL device facilitates the processing of samples without external contact, thereby ensuring the prevention of contamination during the nucleic acid extraction process. Furthermore, the device can extract nucleic acids from up to 4 mL of sample in 30 min by enriching pathogens. The procedure is straightforward and requires only four basic steps: enrichment, lysis, washing, and elution. The extracted DNA was used to simultaneously detect two genes of *O. tsutsugamushi*, thereby enhancing sensitivity compared to detecting a single target gene. The clinical utility was then validated using samples from 38 patients. The specificity and sensitivity were 100% and 95.24% when using a single target gene, but the sensitivity and specificity were 100% when using both target genes. This scrub typhus diagnostic system is more sensitive than existing tests and can be developed into a point-of-care diagnostic system with further research on the detection method.

## 2. Materials and Methods

### 2.1. Chemicals and Reagents

3-aminopropyl(diethoxy)methylsilane (APDMS, 97%), Hyflo Super Cel (Diatomaceous earth), dimethyl suberimidate dihydrochloride (DMS, 98%), sodium hydroxide solution (50% in H_2_O), sodium citrate, 10 mM EDTA, and Triton X-100 were purchased from Sigma-Aldrich (St. Louis, MO, USA). Phosphate-buffered saline (PBS; 1x, pH 7.4) was purchased from GeneAll (Daejeon, Republic of Korea). Distilled water (DNase/RNase-Free), Tris-HCI (pH 8.0), and EDTA (pH 8.0) were purchased from Infusion tech (Seoul, Republic of Korea). Absolute ethanol was purchased from Merck (Whitehouse Station, NJ, USA).

### 2.2. Preparation of Amine-Functionalized Diatomaceous Earth Using I-PULL Device

The I-PULL device is designed to simplify nucleic acid extraction from large sample volumes, up to 5 mL without the need for electricity. The device can be used with previously reported amine-functionalized diatomaceous earth (ADE) [19,20] to reduce the overall time for nucleic acid extraction. The procedure for the preparation of diatomaceous earth can be summarized as follows: 2 mL of APDMS (Sigma-Aldrich, 371890) was added dropwise into 100 mL of 95% ethanol acidified with acetic acid (pH 5.0); 0.5 g of DE (Sigma-Aldrich, 56678) was extensively washed with distilled water (DW), filtered using a sieve to collect particles smaller than 20 μm, and resuspended with a mixture of APDMS and 95% acidified ethanol. After incubation at ambient temperature for 4 h under powerful stirring, functionalized DE was washed with ethanol and DW, dried, and resuspended with appropriate buffer for stability.

### 2.3. DNA Extraction and Detection Using the I-PULL Scrub Typhus Diagnostic System

The procedure for DNA extraction using the I-PULL device was as follows: (1) Nucleic acid extract from the enriched pathogen solution with plasma and buffy coat mixture were added to 1 mL of lysis buffer (100 mM Tris-HCl (pH 8.0), 10 mM EDTA, 1% SDS, 10% Triton X-100), 200 μL of DMS (25 mg/mL), and 200 μL of ADE (40 mg/mL) in an I-PULL device container. (2) The container lid was closed, the mixture was incubated for 10 min, and the handle was pulled downward. (3) Afterwards, the PTFE filter was washed with 2 mL of PBS. (4) The PTFE filter was then removed from the I-PULL and the nucleic acid was extracted from the ADE using elution buffer. The isolated DNA was used to diagnose scrub typhus using the scrub typhus qPCR kit. The qPCR cycling conditions were as follows: an initial denaturation step at 95 °C for 2 min; 45 cycles at 95 °C for 30 s; and 65 °C for 30 s. Amplification reactions containing 5 μL of DNA were performed with a Taq man probe method Primera^TM^ Scrub Typhus Real-Time PCR Detection Kit (Infusion tech, Seoul, Republic of Korea, Cat No. PMD021) in a CFX96 Touch Real-Time PCR Detection System (Bio-Rad, Hercules, CA, USA) in accordance with the manufacturer’s instructions.

### 2.4. Clinical Specimens

All adult patients with suspected scrub typhus who visited Pusan National Yangsan hospital from July 2022 to December 2023 were enrolled prospectively. Scrub typhus was defined by the presence of (1) acute onset fever; (2) at least one of these symptoms or signs, including skin rash, eschar, headache, thrombocytopenia, elevated liver enzymes, lymphadenopathy, hepatosplenomegaly, or pleural effusion; and (3) positive results obtained with real-time PCR targeting the *O. tsutsugamushi*-specific 16S rRNA gene or four-fold increase in *O. tsutsugamushi*-specific antibody titers by immunofluorescence assay (IFA) testing between 2 separate samples obtained in the acute phase and convalescent phases. We assigned patients to the non-scrub-typhus group when alternative clinical diagnoses were established with negative results of real-time PCR targeting the *O. tsutsugamushi*-specific 16S rRNA gene. Additionally, patients without a definitive diagnosis who had negative results in *O. tsutsugamushi*-specific antibody tests (total immunoglobulin titer < 1:40 in IFA) of two samples obtained from the acute and convalescent phases, as well as negative results of real-time PCR targeting the *O. tsutsugamushi*-specific 16S rRNA gene, were included in the non-scrub-typhus group. This study was approved by the Institutional Review Board of the Pusan National University Yangsan Hospital (04-2022-028) and was provided with biospecimens and clinical data from the Institutional Biobank Project (OF-2022-16) according to the individual research protocol. Informed written consent was obtained from the patients.

## 3. Results and Discussion

### 3.1. Principle of Scrub Typhus Diagnosis Using Pathogen Enrichment and Nucleic Acid Extraction Device

**Figure 1:**
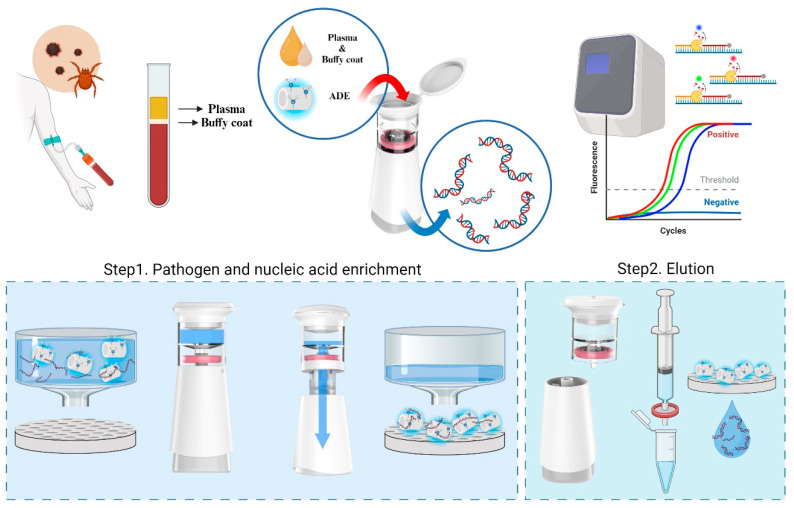
Schematic diagram of rapid pathogen diagnostic systems using I-PULL device. In situ sample processing involves pathogen enrichment and DNA extraction from plasma and buffy coats using amine-functionalized diatomaceous earth within 40 min. Detection for scrub typhus DNA used another gene. This novel system can be used for the rapid, simple and sensitive diagnosis of scrub typhus. The nucleic acid extraction step using the I-PULL instrument is divided into two main steps. The first step is the concentration of the pathogen and the binding of the nucleic acid to the surface of the ADE. The whole process takes place inside a sealed instrument, safe from external contamination. The solution in the container can be passed through the PTFE membrane by pulling down on the handle. Next, the filter membrane is removed, and the elution solution is injected to separate the nucleic acids.

The process of the scrub typhus molecular diagnostic system is divided into two distinct phases: sample preparation and detection (Figure 1). Initially, blood samples are collected from patients, and plasma and buffy coat are separated by centrifugation. Next, amine-functionalized diatomaceous earth (ADE), dimethyl suberimidate dihydrochloride (DMS), and lysis buffer are placed in an I-PULL container along with the mixed plasma and buffy coat samples and incubated for 5–10 min at room temperature. DMS, having two amine groups, binds to nucleic acids isolated from pathogens, while the amine group on the other side binds to the surface of the ADE through electrostatic interaction. Since I-PULL containers can hold volumes up to 10 mL, increasing the amounts of ADE and DMS in a specific ratio allows for the extraction of nucleic acids from large volumes of plasma and buffy coat mixtures. As a result, large sample volumes can be pre-processed to enhance diagnostic specificity and sensitivity. The container, filter membrane, pump, and waste-box fluid reservoir are all hermetically sealed to protect against external contamination. Furthermore, the I-PULL includes a hand-operated pump, enabling nucleic acid extraction without the need for additional equipment. The solution in the container can be passed through the PTFE membrane by pulling down on the handle. Consequently, ADE, which is larger than the pore size, will be collected on the membrane, while other unwanted materials will be directed to the waste box (Step 1). Following the removal of superfluous material through the washing process, the PTFE membrane is removed from the I-PULL device. Elution buffer (pH 10) is then used to elute the DNA and ADE (Step 2). The extracted DNA is confirmed using a developed multiplex quantitative PCR kit.

### 3.2. System Design and Characterization of I-PULL

**Figure 2:**
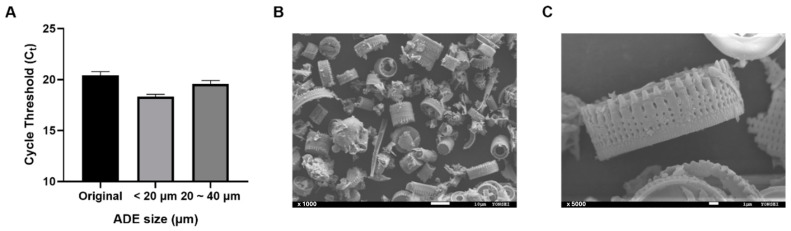
Optimization and characterization of amine-functionalized diatomaceous earth (ADE). (**A**) Comparison of nucleic acid extraction efficiency and ADE size. (**B**,**C**) SEM images of 20 um size and sieved ADE.

To enhance the efficacy of nucleic acid extraction, we undertook the process of homogenizing the size of the ADE, which was sieved into different size fractions, to obtain a homogeneous sample of a single size. A comparison of the nucleic acid extraction efficiency using different sizes of ADE revealed that the 20 µm size exhibited the highest extraction efficiency (Figure 2A). The SEM images showed that the 20 µm ADE retained its cylindrical shape most effectively (Figure 2B,C), indicating it possesses a larger surface area for the same volume than its non-cylindrical form, thereby enhancing its capacity to bind nucleic acids.

**Figure 3:**
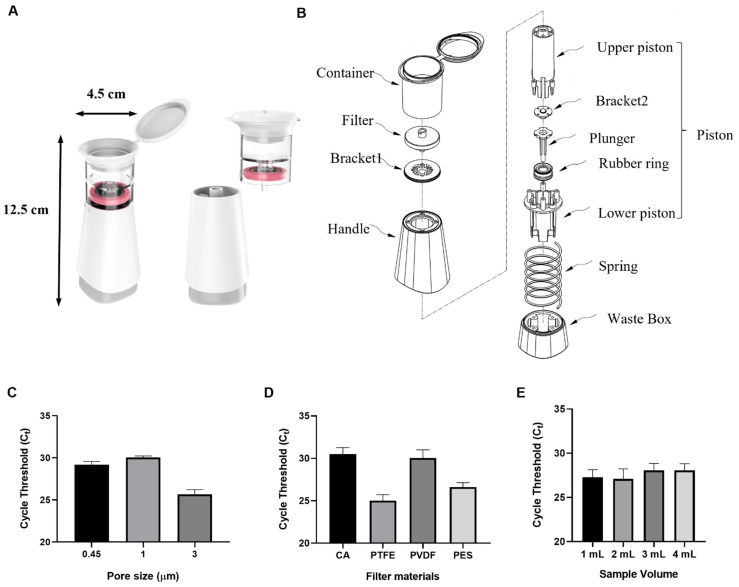
Characterization of the I-PULL device. (**A**) I-PULL device 3D illustration. The dimensions of the device are 12.5 cm high by 4.5 cm wide. The device consists of a container and a body. (**B**) Schematic of the I-PULL device. The I-PULL is divided into three main parts. (**C**,**D**) Selection of filter pore size and material used in the device. (**E**) Comparison of pathogen enrichment efficiency of the same concentration in different PBS volumes.

The I-PULL device was developed for pathogen enrichment and nucleic acid extraction methods without the need for other equipment. The device consists of three parts: the part where the sample is placed, the body part that acts as a handle, and the internal piston that generates pressure to move the solution downward (Figure 3). To generate pressure in response to the movement of the handle, the piston is located inside a spring. In Figure 3, the piston consists of an upper piston and a lower piston. The plunger, with a rubber ring bonded to the top, is screwed to the bottom of the inside of the lower piston. Bracket 2, which prevents backflow of the solution, is fixed to the top of the inner part of the upper piston. The lower and upper pistons are combined and screwed to the handle and waste box, respectively. The spring is clamped between the waste box and Bracket 1, which is attached to the handle. When the handle is pushed down, the piston moves downward with it, creating pressure, and is returned to its original position by the spring. There is a filter at the bottom inside the container, which is seamlessly connected to Bracket 1. Therefore, samples with DNA to be extracted, such as blood, urine, and lysate, in the container will flow down when the handle is pushed down. The device mainly consists of a container for sample injection, a PTFE filter, a handle, and a waste box. It measures 12.5 cm in height by 4 cm in width and can be easily used with one hand. When using ADE, impurities may not be removed properly depending on the filter material or pore size, so we optimized the membrane material and pore size of the filter used. All optimization experiments were performed with the same concentration of *E. coli* (10^5^ CFU/mL) in human plasma, and the highest efficiency was obtained with a hydrophobic 3 µm pore size PTFE filter (Figure 3C,D). In Figure 3E, we compared the enrichment efficiency by diluting the same concentration of *E. coli* in human plasma at different volumes. To avoid blocking the filter due to the high viscosity of the plasma and suspended solids, the plasma and PBS were diluted in a 1:1 ratio and concentrated using ADE and DMS for 15 min at room temperature. The results showed that the I-PULL device was capable of pathogen enrichment and nucleic acid extraction at volumes up to 4 mL. Particularly, the I-PULL device was also developed for single-use only and was entirely isolated from the external environment, ensuring safety from cross-contamination and infection.

### 3.3. Optimization of Nucleic Acid Extraction Using the I-PULL Device

**Figure 4:**
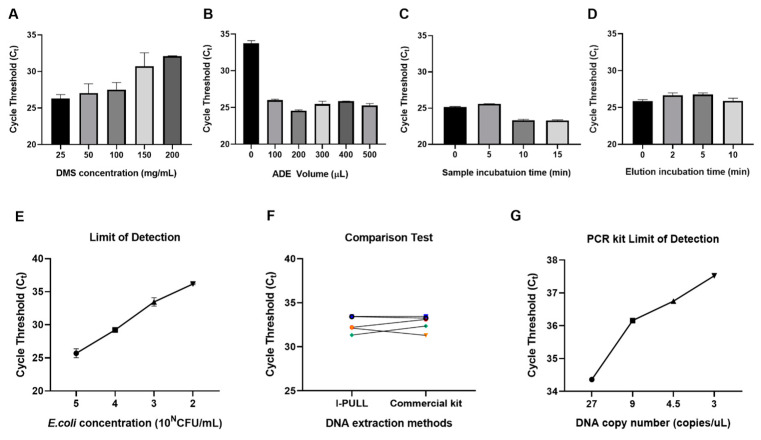
Optimization of nucleic acid extraction and PCR kit detection efficiency using the I-PULL system. The DNA extraction process was optimized and evaluated based on the cycle threshold (C_t_) values after performing real-time quantitative PCR (qPCR). (**A**–**D**) Optimization of DNA extraction conditions. Results as a function of (**A**) DMS concentration and (**B**) ADE volume for DNA extraction. (**C**) Optimization of incubation time and (**D**) elution time for binding of ADE to DNA. (**E**) Limits of detection for DNA extraction based on optimizations results. (**F**) Comparison experiment of DNA extraction using patient samples. Plasma and buffy coat from five scrub typhus patients were used to extract DNA using two different methods (I-PULL and a commercial kit). Each patient was represented by a different color. (**G**) A serial dilution of synthetic DNA was prepared. The amplification plot is represented.

The optimization of the nucleic acid extraction process using I-PULL was performed using qPCR Cycle threshold (Ct) values. We evaluated the binding efficiency of ADE and DNA by using different concentrations of DMS (Figure 4A). In a previous study, we found that an excessive DMS concentration negatively affected the detection process [21,22,23]. Therefore, we examined different concentrations of DMS during nucleic acid extraction using I-PULL. The lowest Ct value was achieved at a DMS concentration of 25 mg/mL (Figure 4A). Using an amount of ADE that exceeds the capacity of the PTFE filter can clog and potentially damage the filter. If the filter is damaged, ADE can pass through, reducing the efficiency of nucleic acid extraction. The filter may also become blocked during the washing process, leading to inadequate cleaning. Therefore, optimizing the amount of ADE is necessary (Figure 4B). ADE was added in varying volumes at a concentration of 40 mg/mL. The lowest Ct value was obtained when a volume of 200 µL was used. Based on the optimization results, we selected a 25 mg/mL DMS solution and 200 µL of ADE (at 40 mg/mL concentration) for the subsequent process (Figure 4C). To enable rapid diagnosis, the sample incubation time and elution incubation time were evaluated to determine the optimal conditions for DNA extraction (Figure 4D,E). The results indicated that the sample incubation time of 10 min was sufficient for binding DNA. The nucleic acid is cross-linked to DMS on the surface of the ADE. Previous studies have confirmed that nucleic acids bind to DMS through electrostatic interactions and amide bonds. To break these bonds, high-pH elution buffers are used [21,22,23,24]. We found that the elution buffer can break the binding instantaneously, with no incubation time required (Figure 4D). DNA isolated from the surface of the ADE passes through the PTFE membrane, while the ADE remains on the filter. We evaluated the performance of I-PULL as a nucleic acid extraction method using serially diluted samples of E. coli ranging from 10^5^ to 10^2^ CFU/mL in plasma (Figure 4E). A comparative experiment was conducted by extracting nucleic acid from plasma and buffy coat samples from five scrub typhus patients between the I-PULL device and a commercial extraction kit (Figure 4F). The results demonstrated that the I-PULL method had comparable efficiency to the commercially available kit. However, the I-PULL method was able to extract nucleic acids within 30 min without any equipment, which could be used as a nucleic acid extraction device for the detection of various diseases as a point-of-care diagnosis in the future.

### 3.4. A Quantitative PCR Kit for Multiplex Genome Detection of O. tsutsugamushi

Since the genome of O. tsutsugamushi exhibits high genetic polymorphism [25], simultaneously detecting or identifying multiple molecular targets in O. tsutsugamushi helps increase the accuracy of a molecular diagnostic tool. Even with proper design of primers and probes and appropriate selection of target locations for amplification, genetic variation in unspecified regions can lead to insufficient amplification. If a positive diagnosis is made when even a single target out of several is amplified, the probability of a false negative can be reduced, which is clinically important. For these reasons, we developed the Primera^TM^ Scrub Typhus Real-Time PCR Detection Kit, which is based on a multiplex real-time PCR assay using TaqMan probes targeting the genes encoding the 47 kDa antigen and the GroEL protein. To evaluate the performance of the kit, we examined the limit of detection using synthetic gene fragments, in which the regions containing the amplified sequence for each target are arranged consecutively (Figure 4G). According to the PCR results from the serial dilution of concentrated gene fragments, the kit consistently detected at least 3 copies/µL of fragments.

### 3.5. Clinical Utility of the Scrub Typhus Diagnostic System on Clinical Samples

**Figure 5:**
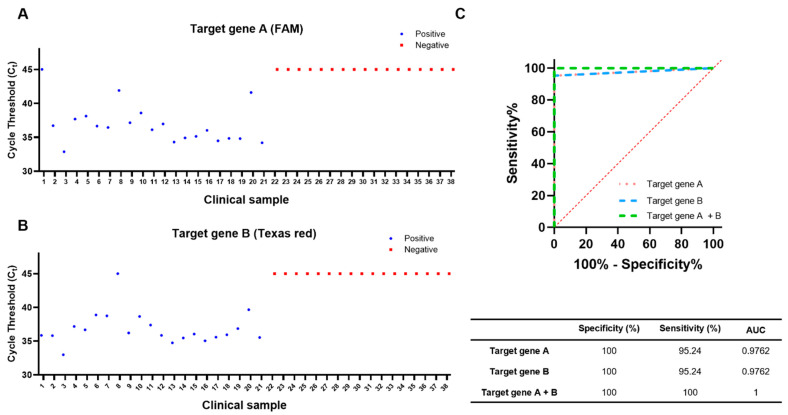
Clinical application of the scrub typhus diagnostic system with plasma and buffy coat. Plasma and buffy coat isolated from 38 different febrile patients were analyzed by nucleic acid extraction using I-PULL device followed by qPCR. (**A**) Comparison of Ct values of 21 scrub-typhus-positive patients and 17 negative patients for target gene A and target gene B (**B**). (**C**) The ROC graph shows the results of using target genes A and B individually and the results of using both target genes together. The diagnostic result using the two target genes is 100% specificity and sensitivity.

To validate the clinical utility of the scrub typhus diagnostic system, 38 blood samples from different febrile patients were tested. The plasma and buffy coat mixtures were analyzed using the scrub typhus diagnostic system, which consists of a sample preparation step (I-PULL nucleic acid extraction device) and a DNA detection step (quantitative PCR). A total of 38 patients with suspected scrub typhus were enrolled in this study: 21 were diagnosed with scrub typhus, and 17 were assigned to the non-scrub-typhus group (Figure 5). The non-scrub-typhus group exhibited bacterial infections (n = 5, 29%), including bacteremia (n = 4) and urinary tract infection (n = 1); SFTS (n = 2, 12%); systemic lupus erythematosus (n = 1, 6%); Guillain–Barré syndrome (n = 1, 6%); and no definitive diagnosis based on molecular and serological test results (n = 8, 47%). The clinical characteristics of the 21 patients in the scrub typhus group and the 17 in the non-scrub-typhus group are shown in Table 1. Patients in the scrub typhus group had a significantly higher incidence of eschar (81% vs. 12%, *p* < 0.001) and skin rash (14% vs. 4%, *p* = 0.008) compared to those in the non-scrub-typhus group. Of 21 patients with scrub typhus, 20 revealed positive 16s rRNA real-time PCR results, and 13 showed seroconversion or more than a four-fold rise in *O. tsutsugamushi*-specific antibody titers by IFA testing between paired samples. The median time from illness onset to sampling for PCR testing was 5 days (IQR, 2–7) with no significant difference between the scrub typhus and non-scrub-typhus groups [5 days (IQR 3–7) vs. 3 days (IQR 2–5), *p* = 0.20].

Using the samples and the PCR kit, Figure 5A,B show the Ct values obtained for the two target genes (47 kDa gene and groEL gene). Test results were considered negative if the Ct value was above 40 and positive if it was below 40. All tests were performed in duplicate, and a sample was considered positive if either of the two target genes was detected. All results from negative patients were confirmed as negative; however, two of the positive patients tested positive for only one of the target genes, target gene A or target gene B. The specificity and sensitivity for one target gene were 100% and 95.24%, respectively (Figure 5A,B). However, the diagnostic performance of the PCR test using two target genes achieved a sensitivity of 100% ([21/21], 95% confidence interval [CI], 84–100), a specificity of 100% ([17/17], 95% CI, 80–100), a positive predictive value of 100% (95% CI, 84–100), and a negative predictive value of 100% (95% CI, 80–100) (Figure 5).

## 4. Conclusions

In this study, we developed a nucleic acid pretreatment technology that can be used for point-of-care testing (POCT). The I-PULL device is designed to separate nucleic acids using plasma and buffy coat separated from whole blood. It uses ADE to concentrate pathogens and extract nucleic acids and is capable of processing large volumes of samples. Additionally, the up-and-down movement of the internal piston generates pressure, facilitating the movement of the solution. Thus, this device can extract high concentrations of nucleic acids within 30 min without the need for other equipment, and it provides a contamination-free extraction method as it is completely isolated from the external environment. However, it has the disadvantages of having to move the filter in the I-PULL during the DNA elution step and the fact that only one sample can be processed. These disadvantages should be overcome with further research. Detection of *O. tsutsugamushi* is challenging due to high rates of false positives and false negatives, largely stemming from its high genetic polymorphism. To overcome these limitations, quantitative PCR is the optimal detection method for *O. tsutsugamushi*. Our findings demonstrate that detecting two different targets can enhance sensitivity and specificity compared to using a single target, achieving 100% sensitivity and specificity with real patient samples. Several real-time PCR assays targeting the 47 kDa gene, groEL gene, 56 kDa gene, or 16S rRNA gene have been reported previously [13,25,26,27,28,29,30]. The sensitivity of real-time PCR in patients with scrub typhus varies widely, ranging from 47.3% to 91.9% depending on the detection method and study design. However, most real-time PCR-based assays encounter the problem of false-negative results due to the high degree of genetic polymorphisms in the *O. tsutsugamushi* genome [31]. Real-time PCR targeting a single gene can lead to false negative results due to primer–template or probe–template mismatches. Using a multiplex real-time PCR approach can overcome this problem because the presence of multiple binding sites increases the likelihood of detecting the target site. The sensitivity of our multiplex real-time PCR was higher than that of previous single-plex real-time PCR [13,26,27,28,29,30]. Only one study conducted in Thailand has performed multiplex real-time PCR targeting the 47 kDa gene and groEL gene in patients with scrub typhus, reporting a sensitivity of 86.5% in patients who had scrub typhus confirmed by IFA testing [25]. The differences in sensitivity between the two multiplex real-time PCRs could be associated with differences in the distribution of *O. tsutsugamushi* strains between Korea and Thailand [32,33]. However, this is considered relatively unlikely because groEL and 47 kDa are conserved regions in most *O. tsutsugamushi* strains [31,34]. Another reason could be that real-time PCR sensitivity is highly dependent on the level of bacteremia, which is affected by the duration of illness, disease severity, or antibiotic administration [26,30]. Further studies with larger clinical samples from more diverse countries are required to confirm the diagnostic performance of our multiplex real-time PCR assay.

While we have developed a highly sensitive method for diagnosing scrub typhus, a platform that can be used as a point-of-care (POC) diagnostic is still needed. Further research is necessary to develop detection methods suitable for POC diagnosis, in combination with the I-PULL device developed in this study. Based on the detection sensitivity of the two targets, we anticipate developing a method for on-site diagnosis of scrub typhus by combining isothermal amplification methods such as RPA (Recombinase Polymerase Amplification) or LAMP (Loop-Mediated Isothermal Amplification) with LFA (Lateral Flow Assay) and colorimetric detection. Therefore, this molecular diagnostic platform could represent a more sensitive tool for diagnosing scrub typhus, enabling rapid differentiation from other acute febrile diseases and aiding clinical decision-making.

## Figures and Tables

**Table 1:** Clinical characteristics of 21 patients with scrub typhus and 17 patients without scrub typhus.

	Total (n = 38)	Scrub Typhus (n = 21)	No Scrub Typhus (n = 17)	*p* * Value
Age, median (IQR), y	67 (60–73)	64 (58–72)	71 (62–76)	0.089
Male sex	19 (50)	10 (48)	9 (53)	0.74
Exposure to field	22 (58)	13 (62)	9 (53)	0.58
Underlying disease ^†^	20 (53)	10 (48)	10 (59)	0.49
Clinical symptoms				
Eschar	19 (50)	17 (81)	2 (12)	<0.001
Skin rash	18 (47)	14 (67)	4 (24)	0.008
Headache	10 (26)	8 (38)	2 (12)	0.14
Lymphadenopathy	10 (26)	8 (38)	2 (12)	0.14
Hepatomegaly	4 (11)	2 (10)	2 (12)	0.99
Splenomegaly	6 (16)	4 (19)	2 (12)	0.67
Pleural effusion	10 (26)	4 (19)	6 (35)	0.29
Laboratory findings ^‡^				
Thrombocytopenia	25 (66)	15 (71)	10 (59)	0.42
Increased liver enzyme	22 (58)	13 (62)	9 (53)	0.58
Mortality	1 (3)	0 (0)	1 (6)	0.45

Data are the number (%) of patients unless otherwise indicated. Abbreviation: AST, aspartate transferase; ALT, alanine transferase; ICU, intensive care unit; IQR. * Student’s *t*-test or Fisher’s exact test. *p* values < 0.05 were considered to be significant. ^†^ Underlying disease included diabetes mellitus, chronic respiratory disease, chronic renal failure, congestive heart failure, liver cirrhosis, malignancy, or those receiving immunosuppressive treatment. ^‡^ Thrombocytopenia was defined as a platelet count less than 140,000/mm^3^. Increased liver enzymes were defined as a serum AST or ALT > 1.5-fold the upper limit of normal.

## Data Availability

The data that support the findings of this study are available from the corresponding author upon reasonable request.
